# Efficacy and safety of combination of mirabegron and solifenacin in patients with double-J stent related overactive bladder: a prospective study

**DOI:** 10.1038/s41598-022-23795-5

**Published:** 2022-11-07

**Authors:** Qing-lai Tang, Shuang Zhou, Yi-qing Liu, Jie Wu, Rong-zhen Tao

**Affiliations:** 1 Department of Urology, The Affiliated Jiangning Hospital with Nanjing Medical University, Nanjing, 211100 Jiangsu China; 2 Department of Urology, Guanyun People’s Hospital, Guanyun, Jiangsu China

**Keywords:** Drug discovery, Urology

## Abstract

To observe the efficacy and safety of solifenacin and/or mirabegron as a medical expulsive therapy (MET) in patients with double-J stent-related overactive bladder (OAB) symptoms. A total of 219 patients with double-J stent-related OAB symptoms were prospectively randomized into two groups. One-hundred and nine cases in the combination group accepted mirabegron and solifenacin therapy and 110 cases as control only accepted solifenacin therapy. The lower urinary tract symptoms and overactive bladder questionnaire (OAB-q) health-related quality of life (HRQol) and symptom bother score between two groups were compared at the 1st, 2nd and 4th week ends. All of 219 patients were randomly assigned to two groups, of which 109 patients were included in the combination group and 110 in the solifenacin group. The incidences of LUTS, including urgency, frequent urination, and incontinence episodes, in the 2nd week (44.9% vs. 64.5%, *P* = 0.028; 48.6% vs. 62.7%, *P* = 0.036; and 40.4% vs. 56.4%, *P* = 0.018) and the 4th week (14.7% vs. 30.9%, *P* = 0.004; 16.5% vs. 33.6%, *P* = 0.003; and 11.9% vs. 26.4%, *P* = 0.007) after combination treatment were significantly lower than those in the solifenacin group. The incidence of drug-related adverse events in the solifenacin group was higher than that in the combination group, but there was no statistically significant difference (*P* > 0.05). In terms of secondary variables, the OAB-q HRQol score in the combination group was statistically superior in comparison with that in the solifenacin group between the second and fourth week (77.9 vs. 76.4, *P* = 0.020; and 87.9 vs. 85.6, *P* = 0.001). The OAB-q symptom bother score was higher in the solifenacin group than in the combination group (37.6 vs. 36.4, *P* = 0.016; and 26.2 vs. 24.8, *P* = 0.003). Combination therapy of solifenacin and mirabegron demonstrated significant improvements over solifenacin monotherapy in reducing OAB symptoms associated with double-J stents, and providing a higher quality of life without increasing bothersome adverse effects.

## Introduction

A double-J stent, as already known, is most frequently used in endourological practice, and it has gradually becomes an indispensable part of many minimally invasive treatment surgeries, for example ureteroscopic lithotripsy (URL), percutaneous nephrolithotomy (PCNL), and retroperitoneal laparoscopic ureterolithotomy^1^. A double-J stent can be beneficial to patients in relieving ureteral obstruction^2^. However, according to the statistics, approximately 80% of patients may develop stent-related symptoms, such as urinary tract infection (UTI), overactive bladder (OAB) symptoms, stent-related body pain, and hematuria^3,4^. These symptoms, attributed to lower ureteral and bladder spasm because of bladder irritation, are common and have a negative effect on the quality of life, working ability, and sexual activity of both genders^5,6^.

However, previous studies have confirmed that oral drugs, such as tamsulosin (alpha-adrenergic blockers) and solifenacin (muscarinic receptor antagonist), are still the main treatment option to alleviate these stent-related symptoms^7,8^. In 2013, Y. J. Lee reported that postoperative solifenacin use was effective and well-tolerated by patients for the treatment of lower urinary tract symptoms, stent-related body pain, and hematuria irrespective of the gender of patients undergoing ureteroscopy and laser stone fragmentation (URSL) and double-J stent insertion^8^. But many patients developed adverse drug reactions^9,10^. Mirabegron, the first and only selective β3-adrenergic receptor agonist agent, has appeared as an emerging drug class for the treatment of urinary incontinence, urgency, and frequency caused by OAB^11^. Last year, our article showed that mirabegron acts through a different mechanism to facilitate stone expulsion and relieve OAB symptoms with fewer adverse effects^12^.

Currently, there are few studies on the use of mirabegron in patients with double-J stent-related OAB symptoms. Hence, we conducted this prospective, randomized trial to explore new treatment models and assess the effectiveness and safety of solifenacin and/or mirabegron as a medical expulsive therapy (MET) for OAB.

## Methods

### Patients

**Figure 1:**
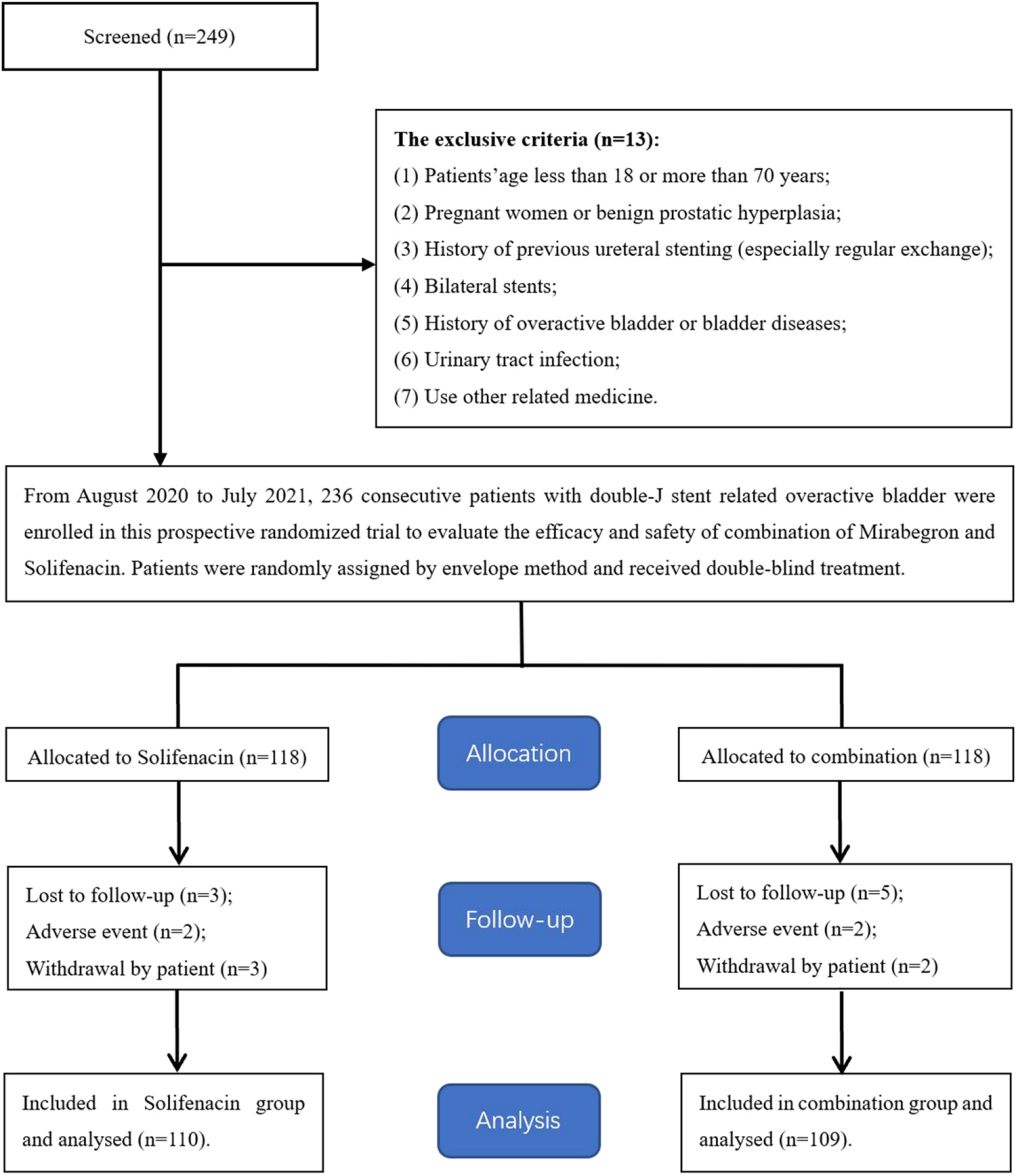
Flowchart for case selection.

From August 2020 to July 2021, eligible patients with double-J stent-related OAB symptoms who were referred to our institute were considered for this study. These patients were divided into the solifenacin group (including 110 patients) and the combination group (including 109 patients) under applying strict inclusion criteria and randomly assigning the patients by the envelope method (Fig. 1). The pre-treatment evaluation data of each participant included general information, medical history, physical examination, laboratory examination and imaging examination. The study was approved by the clinical research ethics committee of the Affiliated Jiangning Hospital of the Nanjing Medical University (ethics approval number: 202100428). Written informed consent was obtained from all participants.

In this prospective study, we evaluated the effectiveness and safety of solifenacin and combination of solifenacin and mirabegron in reducing double-J stent-related OAB symptoms, using the overactive bladder questionnaire (OAB-q) health-related quality of life (HRQol) and symptom bother score.

### Study procedure

The patients in both groups in this study were independent and randomized. Solifenacin 5 mg once a day was given to the patients in the solifenacin group. The other patients in the combination group received mirabegron 50 mg once daily and solifenacin 5 mg once daily. Indomethacin suppository was used as an antispasmodic for intolerable symptom-related OAB. All patients were advised to drink enough water to maintain a daily urine volume more than 1500 ml. Each patient was also asked to complete a real-time questionnaire to report potential drug-related adverse events, and clinical symptoms related to urgency, frequent urination, and incontinence episodes. At the same time, relevant questionnaires were completed by the patients during the follow-up period. The patient can contact the attending doctor at all times if there were any problems related to treatment. If at the end of the follow-up period, the patient's discomfort symptoms have not been relieved, or the remission effect was not satisfactory, relevant further auxiliary treatment will be carried out.

### Follow-up

There was no significant difference at the time of diagnosis and treatment between the two groups. The patients reported the drug-related adverse events each week. Lower urinary tract symptoms (LUTS) at different times was considered as the primary outcome of the study. The secondary end points were OAB-q HRQol score and symptom bother score. Treatment failure was defined as the lack of improvement or relief of LUTS.

### Statistical analysis

SPSS v.22.0 for Windows (IBM Corp., Armonk, NY, USA) was used to perform statistical analysis. Continuous variables were presented as mean ± standard deviation. Patient demographics, follow-up time and clinical outcomes between the two groups were compared using independent samples t test; Chi-squared test was used to compare other clinical characteristics between the two groups. A *P* < 0.05 was considered significant.

### Compliance with ethical standards

All procedures performed in studies involving human participants were in accordance with the ethical standards of Affiliated Jiangning Hospital with Nanjing Medical University and with the 1964 Helsinki Declaration and its later amendments or comparable ethical standards.

### Informed consent

Informed consent was obtained from all individual participants included in the study.

## Results

In this study, 219 patients were randomly assigned to two groups: 110 patients in the solifenacin group and 109 patients in the combination group. The patients’ demographics and clinical characteristics are shown in Table 1. The mean age was 45.3 ± 5.1 years in the solifenacin group and 44.6 ± 6.7 years in the combination group, respectively, and no significant difference was found in the patient age between the two groups (*P* = 0.385). After further study based on the type of OAB, there was still no obvious difference between the two groups. In addition, there was no significant difference between the two groups in body mass index, gender ratio, hypertension, diabetes history, side of the stone, site of the stone, or length of the stent (All *P* > 0.05).

**Table 1:** Comparisons of patients’ demographics and clinical characteristics between two groups.

Variables, mean ± SD or n (%)	Solifenacin group (n = 110)	Combination group (n = 109)	*P* value
Age, year	45.3 ± 5.1	44.6 ± 6.7	0.385
BMI, kg/m^2^	23.7 ± 3.2	24.1 ± 2.7	0.319
**Gender**			
Male	47 (42.7)	53 (48.6)	–
Female	63 (57.3)	56 (51.4)	0.381
**Hypertension history**			
No	69 (62.7)	63 (57.8)	–
Yes	41 (37.3)	46 (42.2)	0.456
**Diabetes history**			
No	72 (65.4)	65 (59.6)	–
Yes	38 (34.6)	44 (40.4)	0.373
**Stone side**			
Right	65 (59.1)	57 (52.3)	–
Left	45 (40.9)	52 (47.7)	0.311
**Stone site**			
Calyceal	14 (12.7)	17 (15.6)	0.543
Pelvis	19 (17.3)	23 (21.1)	0.472
Ureter	77 (70.0)	69 (63.3)	0.293
**Length of stent**			
24 cm	59 (53.6)	48 (44.0)	–
26 cm	51 (46.4)	61 (56.0)	0.155
**Type of OAB**			
Urgency incontinence only	79 (71.8)	73 (67.0)	–
Mixed incontinence	31 (28.2)	36 (33.0)	0.437

*BMI* body mass index, *SD* standard deviation.

*P < 0.05.

Differences in the clinical outcomes between the two groups are shown in Tables 2 and 3. The incidences of LUTS, including urgency, frequent urination, and incontinence episodes, in the 2nd week and the 4th week after combination treatment were significantly lower than those in the solifenacin group (*P* < 0.05). In terms of drug- related adverse events, there was no statistically significant difference between two groups (*P* > 0.05). No other serious complications were noted in this study.

**Table 2:** Comparisons of clinical outcomes between two groups.

Variables, n (%)	Solifenacin group (n = 110)	Combination group (n = 109)	*P* value
**Urgency**			
Baseline	110 (100)	109 (100)	1.000
1st week	92 (83.6)	83 (76.1)	0.167
2nd week	71 (64.5)	49 (44.9)	0.028*
4th week	34 (30.9)	16 (14.7)	0.004**
**Frequent urination**			
Baseline	101 (91.8)	96 (88.1)	0.357
1st week	89 (80.9)	79 (72.5)	0.139
2nd week	69 (62.7)	53 (48.6)	0.036*
4th week	37 (33.6)	18 (16.5)	0.003**
**Incontinence episodes**			
Baseline	94 (85.5)	87 (79.8)	0.271
1st week	81 (73.6)	73 (67.0)	0.280
2nd week	62 (56.4)	44 (40.4)	0.018*
4th week	29 (26.4)	13 (11.9)	0.007**
Drug related adverse events	16 (14.4)	10 (9.2)	0.219
Dry mouth	5 (4.5)	3 (2.8)	–
Tachycardia	2 (1.8)	3 (2.8)	–
Hypertension	3 (2.7)	1 (0.9)	–
Constipation	2 (1.8)	2 (1.8)	–
Dizziness	3 (2.7)	1 (0.9)	–
Urinary retention	1 (0.9)	0 (0.0)	–

*SD* standard deviation;

*P < 0.05, **P < 0.01.

**Table 3:** Comparisons of OAB-symptom score between two groups.

Variables, mean ± SD or n (%)	Solifenacin group (n = 110)	Combination group (n = 109)	*P* value
**OAB-q HRQol score**			
Baseline	59.3 ± 4.7	58.4 ± 3.9	0.125
1st week	63.7 ± 4.1	64.7 ± 4.3	0.080
2nd week	76.4 ± 4.6	77.9 ± 4.8	0.020*
4th week	85.6 ± 4.9	87.9 ± 5.2	0.001**
**OAB-q symptom bother score**			
Baseline	57.2 ± 3.6	58.0 ± 4.2	0.132
1st week	49.5 ± 4.1	48.6 ± 3.9	0.097
2nd week	37.6 ± 3.8	36.4 ± 3.5	0.016*
4th week	26.2 ± 3.3	24.8 ± 3.7	0.003**

*SD* standard deviation, *OAB-q* overactive bladder questionnaire, *HRQol* health-related quality of life.

*P < 0.05, **P < 0.01.

With respect to secondary variables, the OAB-q HRQol score in the combination group was statistically superior in comparison with that in the solifenacin group between the second and fourth week (*P* < 0.05). This was consistent with the primary outcome. On the other hand, From the second to fourth week, the OAB-q symptom bother score was higher in the solifenacin group than in the combination group (*P* < 0.05).

## Discussion

A double-J stent is widely used in urology, but it also causes some stent-related symptoms, including urinary tract irritation, low back pain, and hematuria, which affect the quality of life of patients. It is reported that about 80% of patients with an indwelling ureteral stent have bladder storage symptoms, while about 40% of patients have low back pain symptoms^13^. Currently, various studies are gradually being carried out to improve the material quality and shape of the double-J stent, but the ideal double-J stent has not yet been developed.

**Figure 2:**
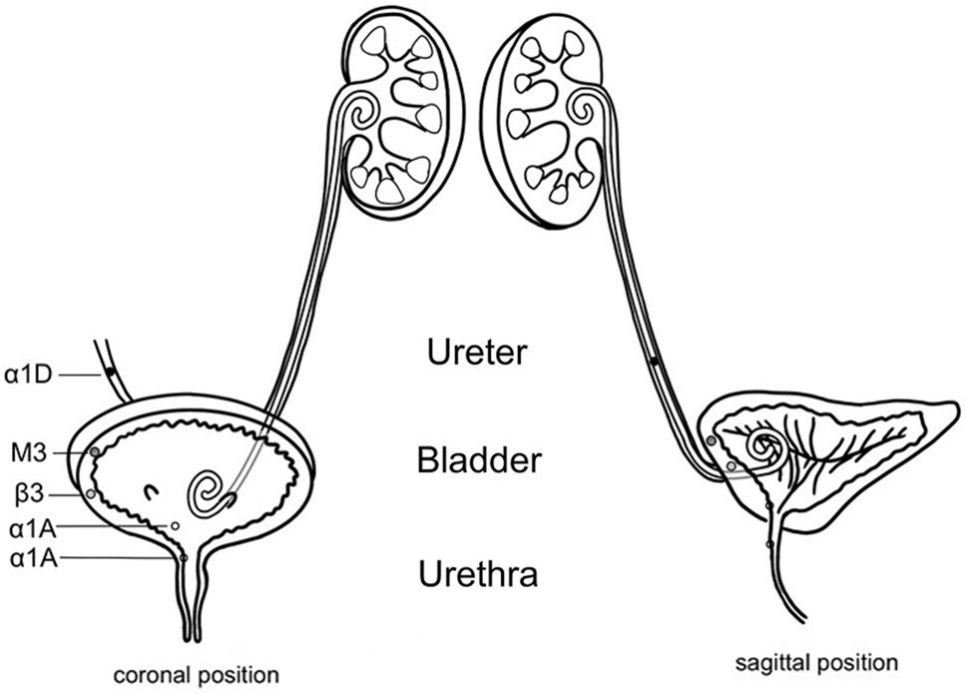
The distribution of various receptors.

The research on drug intervention is mainly based on the distribution of receptors in the bladder. The nervous system mainly affects the activity of bladder smooth muscle through the transmission of neurotransmitters. The essence of this transmission is a type of chemical transmission^14^. Adrenoceptors are widely expressed in the bladder and ureteral tissue (Fig. 2). The M3-receptor is mainly located in the body of the bladder, and its excitation can cause continuous contraction of the detrusor muscle. However, β3 adrenoceptor agonists have appeared as an emerging drug class for the treatment of urinary incontinence, urgency, and frequency caused by OAB^12,15^. The potential role of β3-adrenoceptor agonists in OAB treatment is based on the findings that the human bladder expresses β3-adrenoceptors, and that these β3-adrenoceptors are predominant. Stent-related symptoms may be due to abnormal muscle activity caused by stimulation of the smooth muscle in the bladder by the inner segment of the double-J stent. It mainly presents as a series of clinical manifestations, such as OAB symptoms. Some studies have shown that these symptoms are related to the length of the stent in the bladder; hence, they cannot be fundamentally relieved^16^. Therefore, it is feasible to control the receptor to improve some symptoms during the bladder storage period.

A meta-analysis found that the therapeutic effect of mirabegron on OAB symptoms is similar to that of solifenacin monotherapy, and mirabegron does not increase the risk of adverse events^17^. In terms of combination therapy, several studies have shown that combination therapy of mirabegron and solifenacin can improve OAB symptoms and have similar safety and acceptability as compared with each drug alone and placebo^18–20^. However, currently, there is no study on the application of these two drugs in patients treated with a double-J stent who develop OAB symptoms.

The purpose of our study is to explore the role of a β3-adrenergic receptor agonist agent combined with M3-receptor blocker in improving stent-related symptoms through a prospective randomized controlled study. All patients in the two groups were diagnosed with unilateral urinary calculi. The double-J stent (Bard 5.0 F) with the same diameter was retained after the operation, and the length varied according to the height, so as to eliminate the differences caused by objective factors as much as possible. Our current study showed that the incidences of LUTS, including urgency, frequent urination, and incontinence episodes, in the 2nd week (44.9% vs. 64.5%, *P* = 0.028; 48.6% vs. 62.7%, *P* = 0.036; and 40.4% vs. 56.4%, *P* = 0.018) and the 4th week (14.7% vs. 30.9%, *P* = 0.004; 16.5% vs. 33.6%, *P* = 0.003; and 11.9% vs. 26.4%, *P* = 0.007) after combination treatment were significantly lower than those in the solifenacin group. However, a prospective randomized trial demonstrated no effect of solifenacin in patients with stent-related symptoms^21^. We can infer that mirabegron plays an important role in the combination group to improve the related symptoms. The main acting mechanisms show that mirabegron can stimulate bladder detrusor relaxation and promote urine storage, resulting in an increased bladder volume and prolonged urination interval without affecting bladder emptying. In terms of drug-related adverse events, the incidence in the two groups was similar, and there was no statistically significant difference (*P* > 0.05). No other serious complications were noted in this study. Meanwhile, the OAB-q HRQol score in the combination group was statistically superior in comparison with that in the solifenacin group between the second and fourth week (77.9 vs. 76.4, *P* = 0.020; and 87.9 vs. 85.6, *P* = 0.001). On the other hand, from the second to fourth week, the OAB-q symptom bother score was higher in the solifenacin group than in the combination group (37.6 vs. 36.4, *P* = 0.016; 26.2 vs. 24.8, *P* = 0.003).

However, this study has some limitations. The time for follow-up was short and it may have affected the outcome. Furthermore, we only counted the relevant symptoms through the patient's main complaint, which may have subjective deviation. Finally, the study was based on a single center with a small sample size, and there may be a certain amount of sampling error. Therefore, large-scale multicenter prospective studies are required to further prove the above conclusions. It is likely that the ideal procedure will be formulated through a long period of clinical application and observation.

## Conclusions

Combination therapy of solifenacin and mirabegron demonstrated significant improvements over solifenacin monotherapy in reducing OAB symptoms associated with double-J stents, and providing a higher quality of life without increasing bothersome adverse effects. Combination therapy should be strongly considered for patients who complain of stent-related symptoms. In our opinion, this method is safe and reproducible in clinical practice; however, future prospective clinical studies should include objective physiological measures such as urine flow rate as well as the inclusion of a mirabegron alone treatment group to further prove the above conclusions.

## Data Availability

The datasets used and analysed during the current study available from the corresponding author on reasonable request.
